# The inverse care law in critical care during the COVID-19 pandemic

**DOI:** 10.1016/j.lanepe.2020.100008

**Published:** 2020-12-04

**Authors:** Kirsten J Hainey, Srinivasa Vittal Katikireddi

**Affiliations:** MRC/CSO Social & Public Health Sciences Unit, University of Glasgow, Scotland, United Kingdom

Socioeconomic inequalities in health are pervasive and have been demonstrated across health outcomes and throughout the world. It is perhaps unsurprising that socioeconomic disadvantage is linked to higher risk of SARS-CoV-2 infection [1], but evidence on prognosis after infection remains relatively limited. *Lone and colleagues* used a national cohort to examine the association between living in a socioeconomically deprived area and critical care using timely data linkage [2]. They demonstrate an association between deprivation and both critical care admission and 30-day mortality risk following this.

*Lone and colleagues* provide novel insight into the capacity of critical care units during the first wave of the pandemic in Scotland. Hospitals serving areas of higher deprivation saw greater use of critical care beds compared to hospitals serving areas of lesser deprivation. These findings demonstrate the continued relevance of Tudor Hart's Inverse Care Law, which describes an inverse relationship between the need for healthcare and its availability [3]. Although originally applied in the primary care context, the principles are demonstrated to apply even in the resource-rich context of critical care.

Understanding the socioeconomic gradient in demand for critical care beds is vital for policymakers if they are to mitigate the socioeconomic inequalities in COVID-19 outcomes, as we face a second wave of COVID-19 disease. Proportionate universalism has been advocated in addressing health inequalities, where universal provision of services are combined with targeting distribution to those most in need [4]. *Lone et al's* recent contribution justifies the adoption of such an approach to the resourcing of critical care during the second wave of the pandemic, where additional staff and beds should be prioritised for areas with higher levels of deprivation to meet the greater critical care demand in these areas, whilst ensuring all areas have adequate resource to manage individuals requiring critical care.

Although this research examined the association between COVID-19 outcomes and socioeconomic deprivation in the context of critical illness and intensive care admission, there is growing evidence of a socioeconomic gradient in COVID-19 prognosis across the spectrum of COVID-19 disease severity. Individuals living in more socioeconomically deprived areas are at increased risk of acquiring infection, complications and mortality [1,5]. Understanding the influence of socioeconomic position in COVID-19 and its prognosis is essential in mitigating the population inequalities in both acute and chronic adverse outcomes of infection.

The imminent introduction of effective COVID-19 vaccinations brings hope of controlling the infection and reducing harm. However, the burden of post-acute COVID-19 or ‘Long COVID’ will likely persist as the pandemic resolves. Given the higher incidence of COVID-19 disease in more socioeconomically deprived population, a greater incidence of Long COVID would be anticipated in this group. The inequity in clinical outcomes of COVID-19 disease across the socioeconomic gradient will be compounded by the unequal distribution of the indirect consequences of the pandemic and its impact on individuals’ future health [6].

Whilst *Lone and colleagues* consider the association of socioeconomic deprivation and COVID-19 outcomes, other inequalities were omitted. The high proportion of missing ethnicity data in this cohort exemplifies the challenge of using healthcare records to explore ethnicity, because of their often incomplete or unreliable recording [7]. Equally, examining the role of occupation or individual-level measures of socioeconomic position (such as education level and social class) using healthcare data is hindered by poor recording of such variables in health records in the UK. Data linkage with administrative datasets is necessary to reliably explore the association of these variables with COVID-19 outcomes, and to better understand the contribution of inequalities in risk and clinical outcome of infection.

Inequalities do not exist in isolation and individuals will often experience multiple dimensions of social disadvantage, which will combine to produce their resultant health outcomes. As a result, the importance of applying the lens of intersectionality when examining health inequalities has been increasingly acknowledged in recent years. As an example, the intersection of ethnicity/race and occupation on COVID-19 risk has been demonstrated, where non-White healthcare and essential workers experienced higher infection risks compared to equivalent White workers [8,9]. Whilst exploring the association of socioeconomic inequalities with risk of infection and severity of COVID-19 provides essential information for planning of both health care services and viral suppression measures, understanding the intersection of different dimensions of inequalities will provide a more informed basis for decision-making during the pandemic. Furthermore, understanding the mechanisms underpinning these health inequalities (such as delays in seeking healthcare access or differential quality of care delivered) is an important next step in developing actionable evidence to inform policy.

Scotland has a universal healthcare system which is free at the point of use and prioritises minimising barriers to access. Finding inequalities in this context suggests similar or larger health inequalities will occur elsewhere. Further international research which ideally takes a comparative perspective would be informative, allowing the assessment of if and how inequalities differ across different health systems [10].

## Declaration of Competing Interests

SVK is co-chair of Scottish Government's Expert Reference Group on ethnicity and COVID-19. Except for the funding acknowledged above, we declare no other conflicts of interest.
